# Lentivirus Gene Engineering Protocols

**DOI:** 10.1038/sj.bjc.6601433

**Published:** 2004-01-20

**Authors:** Y Takeuchi

**Affiliations:** 1 University College London, UK

Retroviruses including lentiviruses possess purposeful machinery to insert their genes into host cell chromosomes. Delivering genes of choice in retroviral particles allows their stable and possibly lifelong expression. Traditionally, murine leukaemia virus, an oncovirus but not lentivirus, has been the most studied virus and used for this ‘gene engineering’ purpose. Lentiviruses including human immunodeficiency virus (HIV), deadly human pathogen, have recently attracted much research interest and efforts to take advantage of their property that they, unlike oncoviruses, can efficiently infect nondividing and slowly-dividing cells. This book compiles these 2002-updated efforts in the development and preclinical applications of lentiviral vectors almost exclusively from the gene therapy view point. It is noteworthy that lentiviral vectors are also useful in virological studies and creation of transgenic animals.

Introduction and development of vector systems are dealt with in Part I (seven chapters on the HIV-1 based vector) and Part III (four chapters on non-HIV-1 vectors), while Part II (10 chapters, mainly *ex vivo*) and Part IV (two *in vivo* chapters) describe application examples. All chapters are 10–17 pages, mostly including detailed protocols, so the information in each chapter is limited. It is often unclear what the aims of the experiments are, why lentiviral vectors are useful and what are future problems. It would have been helpful to have more comments on comparison with other vector systems for each application. On the other hand, there is apparent redundancy in technical parts; for example, vector production protocols appear in many chapters, as the editor points out in his Preface. I am not sure how many readers will go through the many, slightly different protocols in the book to appreciate ‘subtle, but significant, differences among different protocols’ unless they have the task of reviewing the book. It is, however, useful to have detailed protocols in each chapter for most readers who pick up specific chapters, which are relevant to their work.

Good overviews as for general knowledge are found in Chapters 2 ‘The choice of a suitable lentivirus vectors’, 6 ‘Detection and selection of lentiviral vector-transduced cells’, 10 ‘Hematopoietic stem and progenitor cells’, 15 ‘Airway epithelia’, 17 ‘Retinal tissue’, 20 ‘FIV vectors’ and 22 ‘Cells of respiratory epithelium’. There is, however, no focus for cancer research. Chapters 8–10 dealing with application on haematopoietic lineages may be relevant for cancer gene therapy; for example, multidrug resistance gene delivery and antigen expression in dendritic cells. Citation of original papers is generally extensive including considerable number of papers published in 2002, but not 2003.

Overall, this book would be useful to have in the laboratory as a quick access to detailed protocols in a variety of gene transfer experiments. Some general, brief information may be more accessible for newcomers and nonexperts than original papers or more extensive review articles. I, however, would not recommend this book as essential reading. One problem is that much of the information in this book may become out of date quickly as many researchers are working hard in this field.

